# 1-Chloro­methyl-1*H*-1,2,3-benzotriazole

**DOI:** 10.1107/S1600536810046052

**Published:** 2010-11-13

**Authors:** Xue-wen Zhu, Ying-Jun Zhang, Chun-Xia Zhang, Gang-Sen Li, Heng-Yu Qian

**Affiliations:** a Department of Chemistry, Zhengzhou University, Zhengzhou 450052, People’s Republic of China; b Key Laboratory of Surface and Interface Science of Henan School of Materials and Chemical Engineering, Zhengzhou University of Light Industry, Zhengzhou 450002, People’s Republic of China

## Abstract

In the title compound, C_7_H_6_ClN_3_, the benzotriazole ring is essentially planar with a maximum deviation of 0.0110 (15)Å, and makes a dihedral angle of 0.46 (8)° with the benzene ring. In the crystal, mol­ecules are linked through inter­molecular C—H⋯N hydrogen bonds, forming chains along the *c* axis.

## Related literature

For bond-length data, see: Alkorta *et al.* (2004 ▶); Wang *et al.* (2008 ▶). For applications of 1-(chloro­meth­yl)benzotriazole, see: Katritzky *et al.* (1996 ▶). For the preparation of the title compound, see: Burckhalter *et al.* (1952 ▶). For the biological activity of benzotriazole derivatives, see: Jiao *et al.* (2005 ▶).
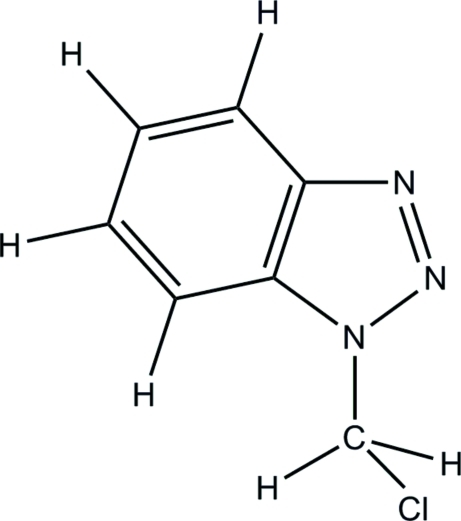


## Experimental

### 

#### Crystal data

C_7_H_6_ClN_3_
*M* *_r_* = 167.60
Monoclinic, 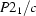

*a* = 7.5081 (17) Å
*b* = 9.6045 (14) Å
*c* = 10.984 (2) Å
β = 108.49 (2)°
*V* = 751.2 (3) Å^3^
*Z* = 4
Mo *K*α radiation
μ = 0.44 mm^−1^
*T* = 293 K
0.21 × 0.20 × 0.19 mm

#### Data collection

Oxford Diffraction Xcalibur Eos Gemini diffractometer
Absorption correction: multi-scan (*SADABS*; Sheldrick, 2004 ▶) *T*_min_ = 0.914, *T*_max_ = 0.922
2865 measured reflections
1530 independent reflections
1218 reflections with *I* > 2σ(*I*)
*R*_int_ = 0.016

#### Refinement

*R*[*F* ^2^ > 2σ(*F* ^2^)] = 0.035
*wR*(*F* ^2^) = 0.088
*S* = 1.06
1530 reflections
100 parameters
H-atom parameters constrained
Δρ_max_ = 0.23 e Å^−3^
Δρ_min_ = −0.16 e Å^−3^

### 

Data collection: *CrysAlis CCD* (Oxford Diffraction, 2010 ▶); cell refinement: *CrysAlis RED* (Oxford Diffraction, 2010 ▶); data reduction: *CrysAlis RED*; program(s) used to solve structure: *SHELXS97* (Sheldrick, 2008 ▶); program(s) used to refine structure: *SHELXL97* (Sheldrick, 2008 ▶); molecular graphics: *SHELXTL* (Sheldrick, 2008 ▶); software used to prepare material for publication: *SHELXTL*.

## Supplementary Material

Crystal structure: contains datablocks I, global. DOI: 10.1107/S1600536810046052/fl2325sup1.cif

Structure factors: contains datablocks I. DOI: 10.1107/S1600536810046052/fl2325Isup2.hkl

Additional supplementary materials:  crystallographic information; 3D view; checkCIF report

## Figures and Tables

**Table 1:** Hydrogen-bond geometry (Å, °)

*D*—H⋯*A*	*D*—H	H⋯*A*	*D*⋯*A*	*D*—H⋯*A*
C7—H7*A*⋯N3^i^	0.97	2.47	3.360 (2)	152

Symmetry code: (i) 
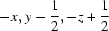
.

**Fig. 1.**
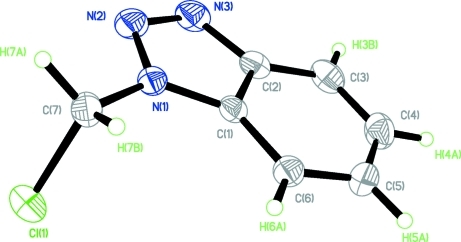
Molecular structure of the title compound showing the atom numbering scheme and displacement dllipsoids drawn at the 30% probability level.

**Fig. 2.**
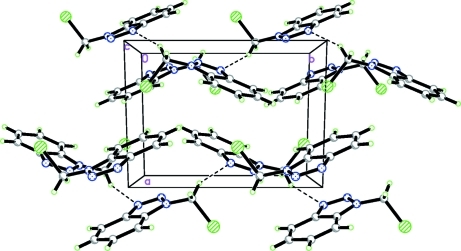
Packing diagram viewed paralled to the c axis. Hydrogen bonds are indicated by dashed lines.

## supplementary crystallographic information

### Comment

Benzotriazole derivatives exhibit a good degree of anti-inflammatory, diuretic and antihypertensive activities (Jiao *et al.*, 2005). The title compound (common name: 1-(chloromethyl)-benzotriazole), as one of the derivatives of benzotriazole, has been synthesized (Burckhalter *et al.*, 1952)and used to synthesize 1-(mercaptomethyl)benzotriazole and other derivates(Katritzky *et al.* 1996). Now, we report herein the crystal structure of the benzotriazole derivative, (I).

The asymmetric unit of (I) comprises of one molecule of the compound (Fig. 1). The bond lengths and angles are found to have normal values (Alkorta *et al*, 2004; Wang *et al.*, 2008). The benzotriazole ring is essentially planar with the maximum deviation form planarity being 0.0110 (15)Å for atom N1. The dihedral angle formed by the ring 1 (N1/N2/N3/C6/C1) and the ring 2 (C1/C2/C3/C4/C5/C6) is 0.46 (8)°. In the chloromethyl group, the C—Cl and C—N bond lengths are 1.7951 (18)Å and 1.424 (2) Å, respectively (Fig. 1). There is a C—H···N intermolecular interaction (Table 1, Fig. 2) stabilizing the observed molecular conformation, and the structure is further stalilized by pi···pi contacts involving both of the aromatic rings (*Cg*(1)—C(*g*)2 = 3.7003 (14) Å, which *Cg*(1) is the centroid of the ring 1 and *Cg*(2) is the centroid of the ring 2).

### Experimental

The title compound was synthesized from 1-hydroxymethylbenzotriazole and thionyl chloride as described in the literature with a yield of 78% (Burckhalter *et al.*, 1952). To 12 g of 1-hydroxymethylbenzotriazole kept at ice-bath temperature, 40 ml of thionyl chloride was added dropwise. The mixture was then stirred and refluxed for 90 minutes. Excess thionyl chloride was removed by distillation, last traces by heating for 15 minutes with 50 ml of methanol. After cooling and collecting on a funnel, the product was then recrystallized from benzene. Crystal suitable for X-ray diffraction analysis was obtained by crystallization from methanol.

### Refinement

H atoms were included in calculated positions and refined as riding atoms with fixed C—H distances [C—H = 0.97Å for CH_2_, and 0.93Å for aromatic CH] and *U*_iso_(H) assigned to 1.2*U*_eq_(C) of their bonding carbon atom.

### Crystal data

**Table:**

C_7_H_6_ClN_3_	*F*(000) = 344
*M**_r_* = 167.60	*D*_x_ = 1.482 Mg m^−^^3^
Monoclinic, *P*2_1_/*c*	Melting point: 409.5 K
Hall symbol: -P 2ybc	Mo *K*α radiation, λ = 0.7107 Å
*a* = 7.5081 (17) Å	Cell parameters from 1327 reflections
*b* = 9.6045 (14) Å	θ = 3.6–26.4°
*c* = 10.984 (2) Å	µ = 0.44 mm^−^^1^
β = 108.49 (2)°	*T* = 293 K
*V* = 751.2 (3) Å^3^	Block, colourless
*Z* = 4	0.21 × 0.20 × 0.19 mm

### Data collection

**Table:**

Oxford Diffraction Xcalibur Eos Gemini diffractometer	1218 reflections with *I* > 2σ(*I*)
Radiation source: Enhance (Mo) X-ray Source	*R*_int_ = 0.016
graphite	θ_max_ = 26.4°, θ_min_ = 3.6°
ω scans	*h* = −9→8
Absorption correction: multi-scan (*SADABS*; Sheldrick, 2004)	*k* = −12→9
*T*_min_ = 0.914, *T*_max_ = 0.922	*l* = −8→13
2865 measured reflections	2865 standard reflections every 0 min
1530 independent reflections	intensity decay: none

### Refinement

**Table:**

Refinement on *F*^2^	Primary atom site location: structure-invariant direct methods
Least-squares matrix: full	Secondary atom site location: difference Fourier map
*R*[*F*^2^ > 2σ(*F*^2^)] = 0.035	Hydrogen site location: inferred from neighbouring sites
*wR*(*F*^2^) = 0.088	H-atom parameters constrained
*S* = 1.06	*w* = 1/[σ^2^(*F*_o_^2^) + (0.0381*P*)^2^ + 0.0967*P*] where *P* = (*F*_o_^2^ + 2*F*_c_^2^)/3
1530 reflections	(Δ/σ)_max_ < 0.001
100 parameters	Δρ_max_ = 0.23 e Å^−^^3^
0 restraints	Δρ_min_ = −0.16 e Å^−^^3^

### Special details

**Table:**

Geometry. All e.s.d.'s (except the e.s.d. in the dihedral angle between two l.s. planes) are estimated using the full covariance matrix. The cell e.s.d.'s are taken into account individually in the estimation of e.s.d.'s in distances, angles and torsion angles; correlations between e.s.d.'s in cell parameters are only used when they are defined by crystal symmetry. An approximate (isotropic) treatment of cell e.s.d.'s is used for estimating e.s.d.'s involving l.s. planes.
Refinement. Refinement of *F*^2^ against ALL reflections. The weighted *R*-factor *wR* and goodness of fit *S* are based on *F*^2^, conventional *R*-factors *R* are based on *F*, with *F* set to zero for negative *F*^2^. The threshold expression of *F*^2^ > σ(*F*^2^) is used only for calculating *R*-factors(gt) *etc*. and is not relevant to the choice of reflections for refinement. *R*-factors based on *F*^2^ are statistically about twice as large as those based on *F*, and *R*- factors based on ALL data will be even larger.

### Fractional atomic coordinates and isotropic or equivalent isotropic displacement parameters (Å^2^)

**Table:**

	*x*	*y*	*z*	*U*_iso_*/*U*_eq_	
Cl1	0.28373 (7)	0.06214 (5)	0.48763 (5)	0.0569 (2)	
N1	0.12629 (19)	0.31134 (15)	0.42214 (12)	0.0387 (3)	
C6	0.2688 (2)	0.44038 (19)	0.63087 (16)	0.0411 (4)	
H6A	0.2580	0.3716	0.6877	0.049*	
N2	0.0960 (2)	0.34462 (18)	0.29614 (13)	0.0509 (4)	
C7	0.0840 (2)	0.17530 (18)	0.45677 (18)	0.0436 (4)	
H7A	−0.0195	0.1369	0.3878	0.052*	
H7B	0.0453	0.1810	0.5329	0.052*	
N3	0.1527 (2)	0.47155 (18)	0.29024 (14)	0.0534 (4)	
C2	0.2235 (2)	0.52323 (19)	0.41342 (16)	0.0403 (4)	
C1	0.2084 (2)	0.42052 (17)	0.49813 (15)	0.0332 (4)	
C4	0.3611 (3)	0.6730 (2)	0.5873 (2)	0.0557 (5)	
H4A	0.4140	0.7581	0.6204	0.067*	
C3	0.3011 (3)	0.6533 (2)	0.4584 (2)	0.0514 (5)	
H3B	0.3111	0.7231	0.4023	0.062*	
C5	0.3451 (3)	0.5681 (2)	0.67153 (19)	0.0504 (5)	
H5A	0.3884	0.5860	0.7592	0.061*	

### Atomic displacement parameters (Å^2^)

**Table:**

	*U*^11^	*U*^22^	*U*^33^	*U*^12^	*U*^13^	*U*^23^
Cl1	0.0606 (3)	0.0432 (3)	0.0670 (4)	0.0114 (2)	0.0204 (3)	0.0072 (2)
N1	0.0447 (8)	0.0379 (8)	0.0319 (7)	0.0053 (6)	0.0098 (6)	0.0008 (6)
C6	0.0460 (10)	0.0417 (10)	0.0365 (9)	0.0045 (8)	0.0142 (8)	0.0023 (8)
N2	0.0602 (10)	0.0574 (11)	0.0318 (8)	0.0121 (8)	0.0099 (7)	0.0023 (7)
C7	0.0432 (10)	0.0380 (10)	0.0489 (10)	0.0000 (8)	0.0135 (8)	−0.0045 (8)
N3	0.0648 (11)	0.0583 (11)	0.0393 (8)	0.0149 (9)	0.0198 (8)	0.0126 (8)
C2	0.0421 (10)	0.0433 (10)	0.0390 (9)	0.0114 (8)	0.0178 (8)	0.0094 (8)
C1	0.0325 (8)	0.0337 (9)	0.0348 (9)	0.0063 (7)	0.0125 (7)	0.0021 (7)
C4	0.0531 (12)	0.0403 (11)	0.0729 (13)	−0.0051 (9)	0.0188 (10)	−0.0092 (11)
C3	0.0534 (12)	0.0393 (11)	0.0682 (13)	0.0027 (9)	0.0286 (10)	0.0137 (10)
C5	0.0539 (11)	0.0513 (12)	0.0432 (10)	0.0019 (9)	0.0114 (9)	−0.0100 (9)

### Geometric parameters (Å, °)

**Table:**

Cl1—C7	1.7950 (18)	C7—H7B	0.9700
N1—C1	1.360 (2)	N3—C2	1.380 (2)
N1—N2	1.3674 (19)	C2—C1	1.385 (2)
N1—C7	1.424 (2)	C2—C3	1.401 (3)
C6—C5	1.367 (3)	C4—C3	1.356 (3)
C6—C1	1.396 (2)	C4—C5	1.399 (3)
C6—H6A	0.9300	C4—H4A	0.9300
N2—N3	1.299 (2)	C3—H3B	0.9300
C7—H7A	0.9700	C5—H5A	0.9300
			
C1—N1—N2	109.78 (14)	N3—C2—C3	130.89 (17)
C1—N1—C7	129.74 (13)	C1—C2—C3	120.79 (16)
N2—N1—C7	120.34 (14)	N1—C1—C2	104.75 (14)
C5—C6—C1	115.31 (17)	N1—C1—C6	132.87 (15)
C5—C6—H6A	122.3	C2—C1—C6	122.38 (16)
C1—C6—H6A	122.3	C3—C4—C5	121.34 (18)
N3—N2—N1	108.53 (14)	C3—C4—H4A	119.3
N1—C7—Cl1	111.25 (12)	C5—C4—H4A	119.3
N1—C7—H7A	109.4	C4—C3—C2	117.14 (17)
Cl1—C7—H7A	109.4	C4—C3—H3B	121.4
N1—C7—H7B	109.4	C2—C3—H3B	121.4
Cl1—C7—H7B	109.4	C6—C5—C4	123.04 (18)
H7A—C7—H7B	108.0	C6—C5—H5A	118.5
N2—N3—C2	108.61 (14)	C4—C5—H5A	118.5
N3—C2—C1	108.32 (16)		
			
C1—N1—N2—N3	−1.26 (19)	N3—C2—C1—N1	−0.84 (18)
C7—N1—N2—N3	−177.32 (15)	C3—C2—C1—N1	179.33 (15)
C1—N1—C7—Cl1	−84.43 (19)	N3—C2—C1—C6	179.77 (15)
N2—N1—C7—Cl1	90.74 (16)	C3—C2—C1—C6	−0.1 (3)
N1—N2—N3—C2	0.7 (2)	C5—C6—C1—N1	−179.59 (17)
N2—N3—C2—C1	0.1 (2)	C5—C6—C1—C2	−0.4 (2)
N2—N3—C2—C3	179.91 (18)	C5—C4—C3—C2	−0.3 (3)
N2—N1—C1—C2	1.27 (18)	N3—C2—C3—C4	−179.37 (18)
C7—N1—C1—C2	176.84 (16)	C1—C2—C3—C4	0.4 (3)
N2—N1—C1—C6	−179.43 (17)	C1—C6—C5—C4	0.5 (3)
C7—N1—C1—C6	−3.9 (3)	C3—C4—C5—C6	−0.2 (3)

### Hydrogen-bond geometry (Å, °)

**Table:**

*D*—H···*A*	*D*—H	H···*A*	*D*···*A*	*D*—H···*A*
C7—H7A···N3^i^	0.97	2.47	3.360 (2)	152

Symmetry codes: (i) −*x*, *y*−1/2, −*z*+1/2.
